# Temperature transcends partner specificity in the symbiosis establishment of a cnidarian

**DOI:** 10.1038/s41396-020-00768-y

**Published:** 2020-09-15

**Authors:** Marcela Herrera, Shannon G. Klein, Sara Campana, Jit Ern Chen, Arun Prasanna, Carlos M. Duarte, Manuel Aranda

**Affiliations:** 1 Red Sea Research Center (RSRC), Biological and Environmental Sciences and Engineering Division (BESE), King Abdullah University of Science and Technology (KAUST), Thuwal, Saudi Arabia; 2 Red Sea Research Center (RSRC) and Computational Bioscience Research Center (CBRC), Biological and Environmental Sciences and Engineering Division (BESE), King Abdullah University of Science and Technology (KAUST), Thuwal, Saudi Arabia; 3 Present Address: Institute for Biodiversity and Ecosystem Dynamics, Faculty of Science, University of Amsterdam, 1090 GE Amsterdam, The Netherlands; 4 Present Address: Department of Biological Sciences, School of Science and Technology, Sunway University, Subang Jaya, Selangor Malaysia

**Keywords:** Climate-change ecology, Animal physiology

## Abstract

Coral reef research has predominantly focused on the effect of temperature on the breakdown of coral-dinoflagellate symbioses. However, less is known about how increasing temperature affects the establishment of new coral-dinoflagellate associations. Inter-partner specificity and environment-dependent colonization are two constraints proposed to limit the acquisition of more heat tolerant symbionts. Here, we investigated the symbiotic dynamics of various photosymbionts in different host genotypes under “optimal” and elevated temperature conditions. To do this, we inoculated symbiont-free polyps of the sea anemone Exaiptasia pallida originating from Hawaii (H2), North Carolina (CC7), and the Red Sea (RS) with the same mixture of native symbiont strains (*Breviolum minutum*, *Symbiodinium linucheae*, *S. microadriaticum*, and a *Breviolum* type from the Red Sea) at 25 and 32 °C, and assessed their ITS2 composition, colonization rates, and PSII photochemical efficiency (*Fv/Fm*). Symbiont communities across thermal conditions differed significantly for all hosts, suggesting that temperature rather than partner specificity had a stronger effect on symbiosis establishment. Overall, we detected higher abundances of more heat resistant Symbiodiniaceae types in the 32 °C treatments. Our data further showed that PSII photophysiology under elevated temperature improved with thermal pre-exposure (i.e., higher *Fv/Fm*), yet, this effect depended on host genotype and was influenced by active feeding as photochemical efficiency dropped in response to food deprivation. These findings highlight the role of temperature and partner fidelity in the establishment and performance of symbiosis and demonstrate the importance of heterotrophy for symbiotic cnidarians to endure and recover from stress.

## Introduction

Heat-induced coral bleaching—the breakdown of a fragile partnership between the coral host and photosynthetic algae of the family Symbiodiniaceae [1]—is a dominant driver of coral reef degradation. Increase in sea surface temperatures by only 1–2 °C above mean summer maxima often causes mass mortality of corals and many other species that depend on the ecosystems these organisms build [2]. However, coral responses to heat stress are not uniform, but instead some species and genotypes resist and/or recover better than others, either because of host genetics [3–7], symbiont identity [8–12], and/or acclimation capacities of the holobiont [13–15]. Particularly, flexibility of the coral host to associate with various photosymbiont strains might be crucial for coping with rapid fluctuations in the environment [16]. Many species can, for example, improve their thermal resilience by temporarily changing their algal symbiont communities (i.e., “shuffling” of preexistent types and/or “switching” to new ones) before, during, and after heat stress events [17–19]. However, this is not a universal feature of coral-Symbiodiniaceae symbioses [20, 21] given that some hosts and symbionts can be less flexible in their association than others. The onset and maintenance of a persistent symbiosis depends on the functional compatibility between the two (or more) partners [22–24] and environmental settings [25–28].

As climate change intensifies and oceans become warmer it becomes increasingly relevant to understand the effect of temperature on the establishment of coral symbiosis. Exposure to elevated temperatures can drastically reduce the initial uptake of symbionts [29] and even impair larvae from establishing symbiosis [30]. Thermal stress can also affect the ability of different taxa to colonize the host such that thermally sensitive symbionts have greater colonization success at lower temperatures and conversely, more heat tolerant types prevail in warmer environments [26, 31]. Given this, it is fair to assume that even if native symbionts adapt to increasing temperatures over time, climate warming might still promote symbioses with already existing heat resistant Symbiodiniaceae strains (e.g., *Durusdinium* sp. [8, 12, 32]) and as a result, change the structure and diversity of host-symbiont assemblages on reefs. Even so, a recent study showed that partner fidelity (i.e., the inherent compatibility between host and symbiont partners) can limit the acquisition of non-native, more thermotolerant symbionts, even under stress conditions [33]. Emerging evidence indicates that coral-Symbiodiniaceae specificity may be a genetically determined trait in some species [34]; and so host-microbe relationships can be highly conserved. This trait has also been documented for coral bacterial communities [35, 36]. Thus, understanding symbiosis specificity is not only important to predict the adaptive evolution of a particular association but also the potential to form new ones, especially in light of future climate change scenarios.

Here, we used *Exaiptasia pallida* [37] (commonly known as Aiptasia) to investigate the extent to which symbiotic associations are host genotype- and temperature-dependent, and how this affects the stress resilience of cnidarian holobionts. We tested the specificity of several Symbiodiniaceae species exhibiting various degrees of heat tolerance [12, 32] in different hosts under “optimal” and elevated temperature conditions. The species were: *Breviolum minitum* which is native to H2 Aiptasia from Hawaii [38], *Symbiodinium linucheae* [39] from North Carolina and symbiotic with CC7 [40], and *S. microadriaticum* and a *Breviolum* strain, isolated from a Red Sea (RS) population [41]. These lineages (host and symbiont) originate from locations with distinct thermal profiles and thus, have likely acquired specific adaptations to their local environments, which is reflected in different stress responses of both partners. In particular, Aiptasia and Symbiodiniaceae taxa from the Red Sea and North Carolina are known to be more thermotolerant than those from Hawaii [39]. Importantly, we selected only symbiont types that are native to our model system (i.e., strains that are naturally found as main symbionts in Aiptasia) and so, we did not perform experiments with the exceptionally heat tolerant strain; *D. trenchii* [8, 12, 32].

We inoculated symbiont-free polyps of all host lines with mixtures comprising equal proportions of the four symbiont strains mentioned above. We assessed the colonization dynamics and PSII photophysiology of these associations among thermal treatments. Based on previous studies [29, 33, 42, 43] in which hosts were simultaneously exposed to multiple symbiont types, we hypothesized that symbionts native to the host line will dominate under “optimal” conditions, but, as temperature increases, more heat tolerant (non-native) types will prevail. In line with evidence showing that pre-exposure to stress improves resilience (reviewed in [44]), we further predicted that the thermal condition in which initial inoculations were performed would have an influence on the holobiont’s response to subsequent elevated temperature. Specifically, we hypothesized that Aiptasia pre-exposed to heat would exhibit greater thermal tolerance than hosts from control, “optimal” conditions, regardless of their genotype and symbiont composition.

## Materials and methods

### Experimental organisms

Aiptasia from three different clonal laboratory strains were used in this study (see Introduction, Table 1). Briefly, anemones were reared in clear polycarbonate containers (2 L capacity; Cambro Camwear, USA) filled with autoclaved natural seawater (~39 psu and pH ~8) at 25 °C under ~40 μmol photons/m^2^s white light on a 12:12 h light:dark cycle (daytime of 06.00–18.00). These light levels, which are similar to those reported by other studies working with Aiptasia [36, 45–48], were chosen to support optimal growth of the animals but also because all symbiont strains used here perform well under this irradiance. Anemones were fed with freshly hatched *Artemia* brine shrimps twice per week, and seawater in the tanks was stagnant and exchanged 2–3 times per week. All populations were maintained under the identical baseline conditions in Percival Scientific Incubators (Model I-22LLVL, USA). Menthol-induced bleaching [49] was used to generate aposymbiotic individuals of each clonal line. Cultures of the strains SSA01 and SSB01 were used to perform inoculations with *Symbiodinium* and *Breviolum* taxa, respectively. Further, Symbiodiniaceae from the Red Sea host line were isolated (Table 1). For a detailed description of the methods refer to Supplementary Methods S1.

**Table 1:** Details of the Symbiodiniaceae cultures used to perform inoculations.

Symbiont strain ID	Aiptasia host source	Original geographic location	Symbiodiniaceae species	Majority ITS2 sequence
SSB01	H2	Hawaii, USA	*Breviolum minutum*	B1
SSA01	CC7	North Carolina, USA	*Symbiodinium linucheae*	A4
RS-A	RS	Al Lith, Saudi Arabia (central Red Sea)	*Symbiodinium microadriaticum*	A1
RS-B	RS	Al Lith, Saudi Arabia (central Red Sea)	*Breviolum* sp.	RS-B1*

*SymPortal revealed different ITS2 type profiles for the Breviolum strains from Hawaii and the Red Sea, thus these were classified as B1 and RS-B1, respectively.

### Effect of temperature on symbiosis establishment

**Fig. 1:**
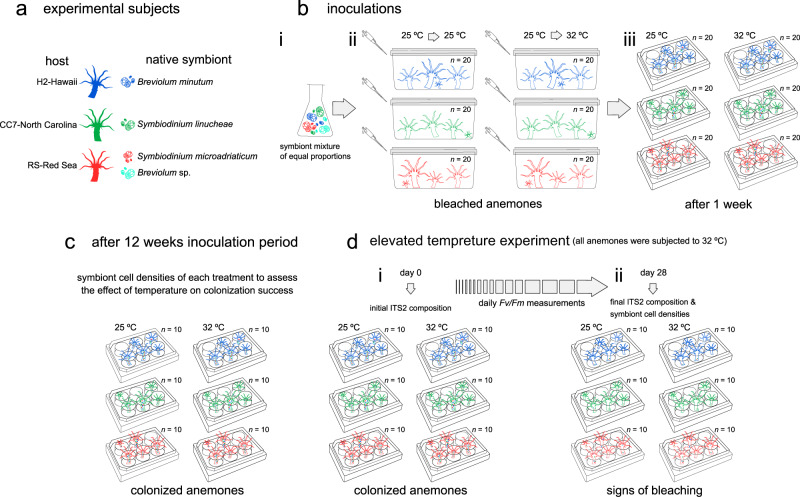
Schematic diagram of the experimental design of this study. **a** Aiptasia host and symbiont strains used to perform inoculations. **b** (i) A symbiont mixture of equal proportions of all four taxa was used to inoculate (long-term acclimated to 25 °C) aposymbiotic anemones from of all three host lineages. (ii) Half of all individuals (*n* = 20 of each genotype) were kept at 25 °C and the rest were transferred to 32 °C. (iii) Polyps were placed in six-well plates (one anemone per well, five anemones per plate). **c** After a 12 weeks inoculation period, symbiont cell densities were assessed for few individuals (*n* = 10) each treatment. **d** The remaining individuals (*n* = 10 per host per temperature) were subjected to 32 °C for 28 consecutive days (25 and 32 °C labels indicate the temperature at which individuals were inoculated). (i) Initial (day 0) and (ii) final (day 28) ITS2 composition were examined, as well as symbiont cell densities.

Inoculations were performed using uniform mixtures containing even proportions of the strains SSA01, SSB01, and two monoclonal cultures that were grown from the previously isolated RS (Red Sea) symbionts (Table 1 and Fig. 1a). Algal strains were grown in axenic cultures for more than 1 year before conducting experiments. Cell density of each culture was assessed using a flow cytometer (BD LSRFortessa, BD Biosciences, USA) to pool equal proportions of each symbiont in the final mixture. In a flow hood, each culture flask was mixed well, and an aliquot was transferred to a 50 mL Falcon tube. The symbiont mixture was centrifuged for 5 min at 3000 × *g*, f/2 media was removed, and cells were re-suspended in 30 mL of autoclaved seawater. The cell suspension was then poured into each 250 mL tank containing the aposymbiotic anemones, followed by feeding with freshly hatched *Artemia* to facilitate the dinoflagellate uptake. Immediately after, half of the tanks were transferred to 32 °C and the others remained at 25 °C (Fig. 1b). The inoculated batches were left overnight, and seawater was exchanged the next day. Inoculations for each treatment (3 hosts × 2 temperatures) were performed separately so each tank (six in total) had at least 20 anemones. The above procedure was repeated three more times over the course of a week, after which polyps were transferred to six-well plates (one anemone per well) (Fig. 1b). In this way, inoculations (of the three hosts) served as independent batches, while the subsequent maintenance of anemones in individual wells provided independent biological replicates.

Inoculations were verified by fluorescence microscopy (Leica DM3000 B inverted phase contrast microscope, Leica Microsystems GmbH, Germany) every day for the first 2 weeks; until color pigmentation was visible to the naked eye. After a 12-week period, when colonization rates were generally stable [49], at least ten polyps from each condition were sacrificed to assess the effect of temperature on colonization success (measured by symbiont cell densities) (Fig. 1c), The remaining individuals (ten anemones of each group) were used in the elevated temperature experiment (Fig. 1d). Noteworthy, some Aiptasia were already acclimated to 32 °C (i.e., 12 weeks inoculation period), thus only anemones that were originally at 25 °C and then transferred to 32 °C can be considered as heat stressed. A tentacle from each individual was taken for DNA extraction to examine the Internal Transcriber Space 2 (ITS2) compositions (at day 0) before subjecting it to 32 °C for 28 consecutive days, during which daily maximum photochemical efficiencies (*Fv/Fm*) (here used as a proxy of thermal response) were recorded with a Pulse Amplitude Modulated fluorometer (Mini-PAM, Walz, Germany). For this, anemones were incubated in darkness for 30 min before taking *Fv/Fm* measurements. After being under elevated temperature for 28 day, tentacles were sampled to assess the ITS2 compositions again, and individuals were analyzed for symbiont counts and protein content (Fig. 1d). In all cases where anemones from 25 °C were transferred to 32 °C, individuals were acclimated over the course of 8 h at increments of 1 °C per h (as previously described in [46]). Five days prior to exposure to elevated temperature, feeding was interrupted but later resumed after day 11.

### Sample processing and ITS2 sequencing

Details on DNA extractions, protein content analysis, and symbiont counts are provided in Supplementary Methods S2. Amplification and sequencing of the ITS2 region was done using the primers (Illumina adapters underlined below) SYM_VAR_5.82S2 (5′TCGTCGGCAGCGTCAG ATGTGTATAAGAGACAG-GAATTGCAGAACTCCGTGAACC 3′) and SYM_VAR_ REV (5′GTCTCGTGGGCTCGGAGATGTGTATAAGAGACAG-CGGGTTCWCTTG TYTGACTTCATGC 3′) [50]. Library preparation is described in detailed in Supplementary Methods S3.

### Identification of Symbiodiniaceae taxa

Sequencing data were analyzed using the SymPortal analytical framework [51]. SymPortal was run locally and all samples, including samples from the isolate symbiont cultures used to perform the inoculations, were analyzed together. Briefly, this software identifies sets of specific ITS2 sequences that re-occur in sufficient numbers of samples and considers them as “defining intra-genomic variants” (DIVs), which are then used to predict an “ITS2 type profile” representative of putative Symbiodiniaceae taxa. However, due to the nature of our data and the availability of pure culture sequence profiles, we used the pre-med file from SymPortal, which represents the sequence associations before the minimum entropy decomposition analyses (i.e., step in which SymPortal consolidates sequences into a smaller set). This approach allowed us to identify the diagnostic (i.e., distinct and most abundant) sequence of each symbiont strain and thus resolve mixed samples in a more accurate manner (see Supplementary Methods S4 and Figs. S1 and S2). Based on this, we identified three main putative taxa: B1, A4, and A1, corresponding to *B. minutum* from Hawaii (SSB01), *S. linucheae* (SSA01), and *S. microadriaticum* (RS-A), respectively. The minor sequence variant 1369_B, on the other hand, was diagnostic of SSB01 as it was not present in the pure Red Sea culture, which we designated RS-B1. The ratio of the minor variant 1369_B to the major B1 sequence thus allowed us to quantitatively resolve the relative abundances of SSB01 and RS-B1 in mixes (see additional details in Supplementary Methods S4 and Table S1). Sequences belonging to other taxa with abundances below 1% were classified as “others”.

### Data analyses

#### Part I: Differences in Symbiodiniaceae communities

All data analyses were conducted in R version 3.5.1 [52] using the package “vegan” [53] to test for differences in the interaction, abundance, and composition of Symbiodiniaceae communities among the fixed and orthogonal explanatory variables; host, temperature, and time point (day). Prior to any analysis, count data (DIVs) were ln (*x* + 1) transformed. Homogeneity of multivariate dispersion (based on Bray-Curtis distances) for each factor was tested with permutation tests of multivariate dispersion (PERMDISP [54]) using the function “betadisper” and set to 9999 permutations. Because the dispersion of samples was significantly different between groups (Table S2), multivariate statistical analyses were conducted per sample type (i.e., separately for H2-Hawaii, CC7-North Carolina, and RS-Red Sea). Two-factorial permutational multivariate analysis of variance [55] were run with 9999 permutations to test for effects of temperature and time in a full-factorial design using the function “adonis”. Differences between treatments were then visualized with principal coordinate analyses plots and further tested using canonical analysis of principal coordinates (CAP) with 9999 permutations [56] using the function “CAPdiscrim” from the “BiodiversityR” package. Finally, negative binomial distribution models (as implemented in DESeq2 [57]) were used to detect differentially abundant DIVs as described previously in [25]. Comparisons included differential abundance testing among relevant temperature and time interactions (Fig. S3). In all cases, *p* values were adjusted with the Benjamini-Hochberg correction method.

#### Part II: Response variables measured

**Fig. 2:**
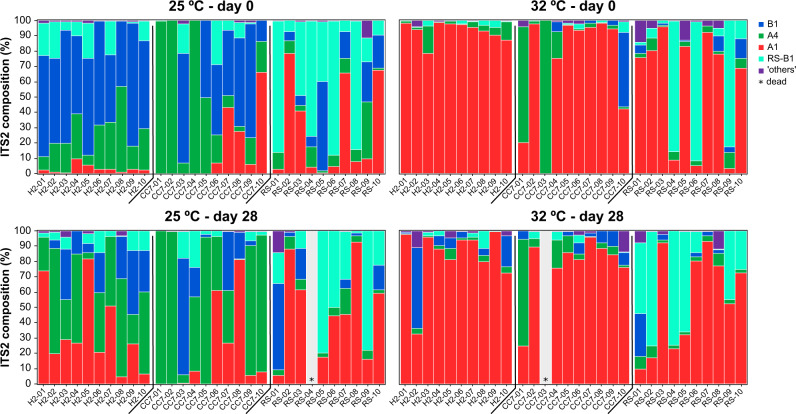
ITS2 composition of the Symbiodiniaceae communities of different Aiptasia colonized at 25 and 32 °C before (day 0) and after (day 28) being subjected to elevated temperature (32 °C). Putative taxa are reported based on their diagnostic sequences and its taxonomic description as referenced in the literature: B1 (*B. minutum* and *B. antillogorgium* and *B. pseudominutum*), A4 (*S. linucheae*), and A1 (*S. microadriaticum*). B1 taxa from the Red Sea isolate are designated as RS-B1. Sequences belonging to other taxa were classified as “others”.

The biological response variables were analyzed using both ANOVAs and repeated linear mixed models (LMMs). All data were first checked for normality and homoscedasticity using standardized residual plots and Q-Q plots and, if required, data were ln (*x* + 1) transformed. Maximum photochemical efficiencies were analyzed using repeated-measures LMMs in SPSS [58]. The fixed factors were host genotype, pre-exposure temperature and time, which was the repeated measure. Due to the unavoidably low and uneven replication among hosts (Fig. 2), symbiont composition was not included as a factor in the analysis. Further, preliminary analyses revealed differences in *Fv/Fm* trends among hosts regardless of their symbiont composition (either at day 0 or day 28). Given that the presence of feeding co-occurred with the factor of time, feeding was not included as a factor, but data were interpreted accordingly. Several repeated covariance types [e.g., AR(1), AR(1) heterogeneous, CS] were investigated to assess the model of best fit by comparing various goodness-of-fit statistics [e.g., -2 restricted log likelihood, Akaike’s information criterion, and Bayesian information criterion]. Estimated marginal means (least-squares means) were used to determine which means differed for the significant, highest-order terms. Finally, symbiont cell densities were analyzed with a two-way ANOVA in R using host and temperature as fixed factors. Tukey pairwise comparisons were further conducted post hoc to determine where significant differences occurred.

## Results

### Temperature effect on symbiont acquisition and maintenance

**Fig. 3:**
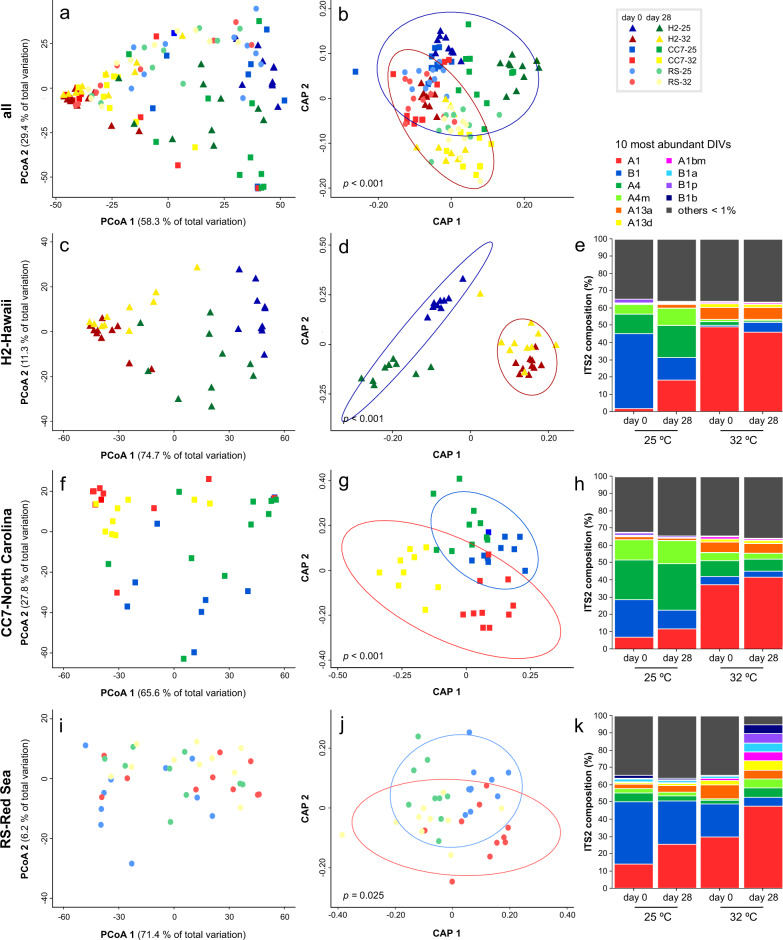
Distinct Symbiodiniaceae communities associated with different Aiptasia hosts at two temperatures after 12 weeks of performing inoculations. Principal coordinate analysis (PCoA) showing variation in the ITS2 composition of the Symbiodiniaceae communities associated with (**a**) all Aiptasia from 25 and 32 °C before (day 0) and after (day 28) being subjected to elevated temperature and for (**c**, **f**, **i**) each host separately. Constrained analysis of the canonical axes of the principal coordinates (CAP) show significant differences (*p* < 0.001) between temperature and time treatments (**b**, **d**, **g**, **j**). Different shades of blue and green represent treatments at 25 °C (day 0 and 28, respectively), whilst red and yellow colors show communities from 32 °C (likewise). Ellipses represent the 95% confidence intervals for the temperature factor (blue for 25 °C and red for 32 °C). Taxonomy bar charts show the relative abundance of the most abundant taxa for each group (**e**, **h**, **k**). All DIVs for which abundance was below 1% were grouped together in the “others” category.

**Fig. 4:**
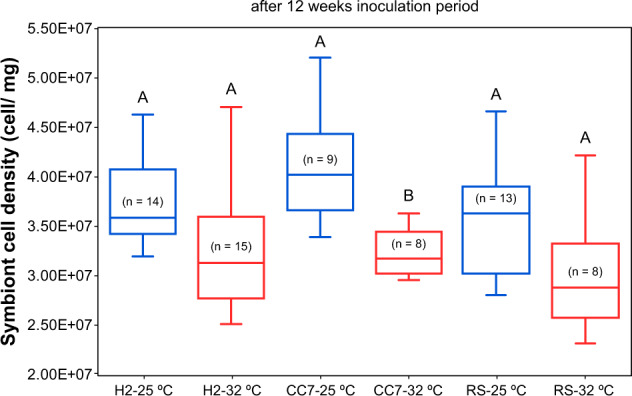
Mean (±1 SE) symbiont cell densities (normalized to protein content) of different Aiptasia from 25 °C (blue) to 32 °C (red) after 12 weeks of performing inoculations. Pairwise comparisons were carried out within each host genotype. Letters above error bars indicate similarities (e.g., AA) or differences (e.g., AB) between host–symbiont combinations, as determined by Tukey’s HSD post hoc test (*p* < 0.05).

Overall, Aiptasia from 25 °C (regardless of the host genotype) were initially (day 0) dominated by *Breviolum* taxa compared to those from 32 °C, which had much higher abundances of A1 (Fig. 2). Symbiodiniaceae communities across all treatments were well segregated (explaining up to 87% of the total variability, Fig. 3a), mainly according to temperature (Fig. 3b). Indeed, significant differences were detected for all groups (Table S3) when analyzed separately (see Fig. 3c, d for H2-Hawaii, Fig. 3f, g for CC7-North Carolina, and Fig. 3i, j for RS-Red Sea). Symbiont composition appeared to differ across time; in particular, anemones from 25 °C showed a substantial increase (up to threefold) of A1 sequences after being exposed to elevated temperature for 28 days (Fig. 3e, h, k). Furthermore, analyses of raw abundances revealed a high number of differentially abundant DIVs across treatments (Table S4). Notably, we detected DIVs associated with *Symbiodinium* (clade A), *Cladocopium* (clade C), and/or *Durusdinium* (clade D). Abundances of these types were significantly higher in communities from 32 °C, particularly at day 0 but also after transferring the 25 °C individuals to this treatment (Fig. S4). Conversely, *Breviolum* (clade B) variants were generally underrepresented in the 32 °C treatment, despite these being the most abundant taxa across all groups. Finally, elevated temperature also had an overall effect on symbiont colonization as evidenced by the lower cell densities in anemones from 32 °C (though this was significantly different for CC7-North Carolina only, Fig. 4).

### Extent of thermal response depends on host genotype and pre-exposure treatment

**Fig. 5:**
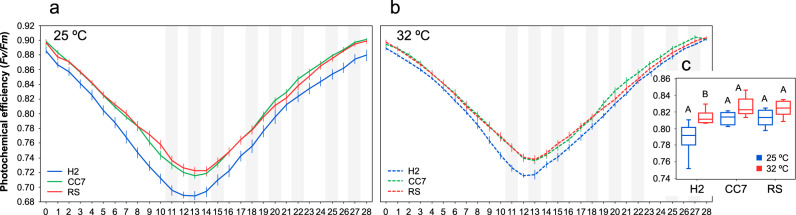
Photophysiological response of different Aiptasia holobionts from two temperatures is affected by food availability. Mean (±1 SE) photochemical efficiencies of different Aiptasia pre-exposed to (**a**) 25 °C and (**b**) 32 °C before (day 0) and throughout 28 days of being subjected to elevated temperature. **c** Overall differences in PSII operating efficiency across groups. Shaded areas indicate days in which animals were fed. Letters indicate overall similarities (e.g., AA) or differences (e.g., AB), as determined by estimated marginal means, between holobionts from different temperatures (25 °C vs 32 °C).

**Fig. 6:**
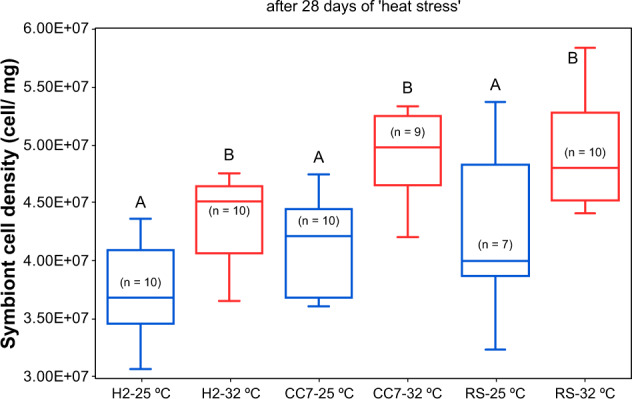
Mean (±1 SE) symbiont cell densities (normalized to protein content) of different Aiptasia from 25 °C (blue) to 32 °C (red) after being subjected to 28 days of elevated temperature. Pairwise comparisons were carried out within each host genotype. Letters above error bars indicate similarities (e.g., AA) or differences (e.g., AB) between host-symbiont combinations, as determined by Tukey’s HSD post hoc test (*p* < 0.05).

We observed a rapid decline in photochemical efficiencies (*Fv/Fm*) from ~0.9 to ~0.7 during the first 10 days of exposure. However, after feeding resumed at day 11, *Fv/Fm* values increased and steadily returned to their initial values by the end of the exposure (day 28) (Fig. 5a, b). Contrary to our expectations, both pre-exposure treatments exhibited similar response patterns (Fig. 5a, b), but the response over time depended on host type, resulting in a significant host × day interaction (*p* < 0.001). Specifically, the same ranking of host responses (RS-Red Sea > CC7-North Carolina > H2-Hawaii) was observed for both treatments (*p* < 0.05), whereby RS-Red Sea consistently exhibited the highest *Fv/Fm* values and H2-Hawaii the lowest. Importantly, however, our analysis detected a significant host × pre-exposure treatment (*p* < 0.001) interaction (Table 2), which resulted from differences between *Fv/Fm* responses of the pre-exposure treatments regardless of time and the inconsistency of this pattern across host types (Fig. 5c). Indeed, within host comparisons revealed an effect of thermal pre-exposure in the response to elevated temperature (Fig. 5c), but this effect was primarily driven by higher photochemical efficiency in the H2-Hawaii individuals from 32 °C compared to their counterparts from 25 °C (*p* < 0.001, Fig. 5c). These data are highly consistent with the significantly greater density of symbionts in pre-exposed hosts after 28 days at 32 °C (Fig. 6).

**Table 2:** Summary of results of a linear mixed-model (LMM) analysis comparing photochemical efficiencies (*Fv/Fm*) of different Aiptasia holobionts that were inoculated at 25 and 32 °C before (day 0) and after (day 28) being subjected to elevated temperature.

Source of variation	Numerator df	Denominator df	*F*	*p*
Host	2	89.742	33.404	*<0.001*
Pre-exposure temp	1	89.742	8.381	*0.041*
Day	28	1146.153	266.155	*<0.001*
**Host** **×** **pre-exposure temp**	2	89.742	10.900	***<0.001***
**Host** **×** **day**	56	1146.153	1.871	***<0.001***
Pre-exposure temp × day	28	1146.153	0.886	0.637
Host × pre-exposure temp × day	56	1146.153	1.089	0.308

Data were Ln (*x* + 1) transformed. The model of best fit was AR(1). AIC (Akaike information criterion) = -11,760.310, BIC (Bayesian information criterion) = -11,749.712. Statistically significant *p* values (0.05) are denoted in italics. In each case, the significant sources of variation are shown in bold.

*df* degrees of freedom.

## Discussion

### Temperature predicts attributes of Symbiodiniaceae communities across treatments

Here, we studied the dynamics of multi-species symbioses establishment in Aiptasia hosts from different regions under “optimal” and elevated temperature conditions using only symbionts that are native to our model system. In accordance with [12, 32], our data are consistent with that symbionts’ thermotolerance followed the order of *S. linucheae* (A4) ≥ *S. microadriaticum* (A1) > *Breviolum* from the Red Sea (RS-B1) > *B. minutum* from Hawaii (B1). Considering previous evidence for symbiont shuffling and/or switching [17, 18], we hypothesized that communities would shift from *Breviolum*-dominated toward more heat tolerant taxa as temperature increased. Indeed, we observed a significantly higher abundance of variants associated with *Symbiodinium* (clade A), *Cladocopium* (clade C), and *Durusdinium* (clade D) taxa in anemones that were pre-exposed to 32 °C, but also in heat stressed individuals (that is, Aiptasia reared at 25 °C and subsequently subjected to 32 °C).

*Symbiodinium* species are known to be more resilient, with lower reactive oxygen species levels and slower deterioration of photosystem (PS) II compared to *Breviolum*, for example [41, 59–62]. Surprisingly, despite *S. linucheae* (A4) being supposedly the most thermally tolerant species tested here (according to [32]), it was barely observed at 32 °C; only in few individuals from 25 °C and for which it was already present in their initial ITS2 composition. Instead, high abundances of *S. microadriaticum* (A1) were detected. We can only presume that in these particular settings, A1 symbionts were more beneficial and/or competitive than A4, at least in the short term. In addition, we expected a shift from B1 to RS-B1 for individuals that were transferred from 25 to 32 °C, however, this was not observed. Moreover, besides having a significant effect in community composition, temperature also moderated the host’s uptake of symbionts. Particularly, and consistent with previous observations [29], lower cell densities (though only significantly different for CC7-North Carolina) were attained in inoculations from 32 °C.

Gabay et al. found strong limitations in the acquisition of thermally tolerant symbionts due to the high specificity of H2-Hawaii Aiptasia (which they refer to as a population from the Indo-Pacific that associates mainly with *B. minutum*). However, considering that they used non-native, foreign symbionts, it is unsurprising that these failed to establish symbiosis [24, 42, 63, 64]. Contrary to the above, we show that (elevated) temperature voids inter-partner fidelity and instead promotes associations with different Symbiodiniaceae. Indeed, various symbiont strains can colonize Aiptasia under laboratory conditions [23, 24, 63, 64], yet, natural populations are known to engage in highly specific symbioses, such that genetic differences in the anemone host correlate with genetic differences in the algal symbiont [65]. Thus, we do not know to what extent environment overrules symbiosis specificity. For instance, despite the strong effect of temperature on symbiont composition, we still observed strain preference to a certain degree, given that H2-Hawaii and RS-Red Sea anemones had more B1 and RS-B1, respectively, at least at 25 °C.

Neither [33] nor our study investigated colonization dynamics beyond a few weeks (4 months in our case), which is extremely important as symbioses are not stable and can change with time. Whether heat tolerant symbionts can be retained over the lifetime of corals remains an open question. More resilient taxa usually come at high energetic costs and reduced growth [22, 66, 67]. Indeed, most adult colonies harbor only one predominant species/genotype, which would be the more beneficial for the host [68], and not necessarily the most thermal tolerant. Predicting the fate of novel partnerships, especially under stress conditions, is not simple. We can only hypothesize that these trade-offs will decrease as temperatures increase, thus promoting the establishment and proliferation of heat resistant symbioses [26].

### Pre-exposure to elevated temperature improves the thermal response of the Aiptasia holobiont

Numerous studies (reviewed in [44]) have demonstrated that the thermal resilience of corals can be improved by rearing juvenile (developmental acclimation) and adult stages (acclimation) at higher temperatures for days or weeks, and/or exposing them to sub-lethal levels of heat stress (hardening) for even just a few hours. Our findings concur with these observations, that is, thermal response (which here we assessed by measuring the PSII operating efficiency) of holobionts preconditioned to elevated temperature improved compared to that of individuals from 25 °C. Specifically, *Fv/Fm* of anemones that were inoculated at 32 °C seemed to be higher than those from 25 °C. However, we also showed that any fitness advantage conferred by temperature-induced acclimation was severely affected by food deprivation, which suggests that the processes involved are energy demanding. Indeed, factors like sex, age, ontogeny, and food availability are known to affect the thermal responses of a wide range of ectotherms [69–71], in addition to inter-individual and species variation.

Elevated temperatures affect both the host and photosymbiont, yet, it has long been suggested that the latter ultimately determines corals’ resilience to stress [8, 9, 11]. Recent work [72] has shown, however, that the host environment (e.g., variations in metabolism) can strongly affect the physiology of even the most heat tolerant symbiont types and thus, alter the overall performance of the holobiont. Indeed, a given host provides specific growth conditions (e.g., in hospite nutrient availability) for the symbiont community which, in turn, affects the productivity of the holobiont system [73]; and so reflects on the ability to (physiologically) compensate for stress in regard to its energetic demands (i.e., environment provided for the symbiont). Our findings support this notion, indicating that thermal response of the Aiptasia holobiont might be fundamentally determined by host genotype. Particularly, CC7-North Carolina and RS-Red Sea have been identified as more (genotypic- and phenotypically) plastic, thermal resistant lineages [39]. Indeed, both CC7-North Carolina and RS-Red Sea (although not different from each other) exhibited higher photochemical efficiencies and greater densities of symbionts than H2-Hawaii regardless of their pre-exposure to elevated temperature. These observations highlight the potential of using Red Sea Aiptasia to study thermal resilience of symbiotic cnidarians, as they thrive in one of the hottest (as high as 35 °C during summer) seas on Earth and naturally experience temperatures predicted elsewhere for the end of the century under “business as usual” emissions pathways without bleaching [74, 75].

### Effect of active feeding during thermal stress

While it was not our intention to specifically test for the effect of food availability, it is not uncommon to interrupt feeding before measuring physiological variables in order to reduce the effect of trophic status [46, 73, 76, 77]. In the present study, animals were unfed prior to (up to 3 days) and partially over the course of the experiment (first 10 days) and so, we cannot disentangle the confounded influence of food availability and temperature. Nevertheless, feeding certainly has a major effect on the response to temperature [78–81]; thus, it is not surprising that Aiptasia from 32 °C also showed signs of stress (as seen by the decline in *Fv/Fm*) when deprived from food. Indeed, previous studies [79, 82] have shown that the photochemical efficiency of fed corals is up to 70% higher compared to starved individuals under thermal stress (but see [83]).

### Methodological considerations

Overall, our results showed that symbiont communities differed substantially among temperature treatments. However, as samples were only taken at the beginning (day 0) and end (day 28) of the experiment, we cannot pinpoint exactly when these changes occurred. It is possible that some individuals were not fully aposymbiotic at the time we performed the inoculations, which could have contributed to the proliferation of symbiont types that were not included in the inoculation mixture. Such was the case for *Cladocopium* and *Durusdinium* taxa that might have been initially present in very low abundances and then increased at elevated temperature (as they are more heat resistant). However, while we cannot exclude potential contamination through incomplete bleaching, feeding (i.e., food was taken from a common batch), and/or subsequently during PCR amplification, our conclusions remain the same; that is, temperature has a greater effect on symbiont composition than partner specificity.

Here, we attributed differences in thermal performance to host genotype and pre-exposure temperature only due to two non-mutually exclusive reasons: (1) low and uneven replication level of most host-symbiont combinations did not allow us to include the symbiont factor in our analyses (i.e., not enough power to detect differences), and (2) we do not know when symbiont communities changed, and if this was the result of elevated temperature alone or if feeding also had an effect. Thus, we are cautious when interpreting our results, as we disregarded any contribution from the symbiont partner but are also unable to disentangle the combined effects of temperature and food deprivation. Nevertheless, our data strongly indicate that symbiont identity played a critical role in moderating the thermal response of the holobionts and thus, their role should not be underestimated. To further elucidate the exact nature of the drivers at play, higher replication of the different host-symbiont combinations across conditions and control treatments (i.e., keeping animals at 25 °C for the 28 days period and with continuous feeding) is likely required. It should be noted that food deprivation can have a significant effect on protein content, which here we used as a reference to normalize symbiont counts. For example, studies [84, 85] have shown that fed corals (even in moderate levels, i.e., twice a week) have up to 49% higher protein content than starved corals. Thus, it is not surprising that symbiont cell/protein ratios differ across experiments such that symbiont cell densities cannot be compared. One final consideration noteworthy of mentioning is that the salinity of the seawater we used to rear all Aiptasia (~39 psu from the central Red Sea) is significantly higher than other studies. The latter is relevant in the context of this study as it has recently been shown that high salinity levels can contribute to greater thermotolerance of cnidarian holobionts [46] by modulating the physiology of its photosymbionts [86].

## Conclusions

Here, we used the flexible coral model *Exaiptasia pallida* and deep sequencing of symbiont communities to assess the establishment and performance of symbioses under different thermal conditions. Our results show that temperature has a significant and greater effect than partner specificity on the symbiont composition of Aiptasia, with increased temperature favoring colonization (and proliferation) of more heat resistant types. Our data add to the body of work showing that pre-exposure to elevated temperature is crucial for the thermal resilience of the cnidarian holobiont, which is also dependent on other factors like, in this case, food availability.

## Supplementary information

Supplemental Information

Supplemental Tables

## Data Availability

Sequencing data are available at NCBI under BioProject ID number PRJNA577392.
