# Supplementary material for: Temperature transcends partner specificity in the symbiosis establishment of a cnidarian
Source: ISME J. 2020 Sep 15;15(1):141–53. doi: 10.1038/s41396-020-00768-y (PMC7852570; doi:10.1038/s41396-020-00768-y)
Supplement: Supplementary file 1 — Supplemental Information [file 41396_2020_768_MOESM1_ESM.docx]

**SUPPLEMENTARY INFORMATION**

**Temperature transcends partner specificity in the symbiosis establishment of a cnidarian**

Marcela Herrera^1^, Shannon G. Klein^2^, Sara Campana^1,3^,

Jit Ern Chen^1,4^, Arun Prasanna^1^, Carlos Duarte^2^ and Manuel Aranda^1*^

^1^King Abdullah University of Science and Technology (KAUST), Red Sea Research Center (RSRC), Biological and Environmental Sciences & Engineering Division (BESE), Thuwal, Saudi Arabia

^2^King Abdullah University of Science and Technology (KAUST), Red Sea Research Center (RSRC) and Computational Bioscience Research Center (CBRC), Biological and Environmental Sciences & Engineering Division (BESE), Thuwal, Saudi Arabia

^3^Present address: University of Amsterdam, Faculty of Science, Institute for Biodiversity and Ecosystem Dynamics, 1090 GE Amsterdam, The Netherlands

^4^Present address: Sunway University, School of Science and Technology, Department of Biological Sciences, Subang Jaya, Selangor, Malaysia

*corresponding author: [manuel.aranda@kaust.edu.sa](mailto:manuel.aranda@kaust.edu.sa)

**Methods S1: Experimental organisms**

Menthol-induced bleaching [1] was used to generate apo-symbiotic individuals of each clonal line. Animals were incubated in autoclaved seawater with 0.19 mmol/L menthol during daytime, followed by a 5 μmol/L 3-(3,4-dichlorophenyl)-1,1-dimethylurea (DCMU) seawater incubation overnight. This treatment was done every other day 4 times a week until complete bleaching was observed and confirmed via fluorescence microscopy (Leica DM3000 B inverted phase contrast microscope, Leica Microsystems GmbH, Germany). Anemones were then kept in a dark incubator for at least two months and further maintained for at least another month on a diurnal 12:12 h light:dark cycle to ensure there was no re-establishment of symbiosis.

Isolate cultures of the SSA01 and SSB01 strains (courtesy of the John Pringle Lab) were used to perform infections with *Symbiodinium* and *Breviolum* taxa, respectively. Further, Symbiodiniaceae from the RS-Red Sea host line were isolated. Briefly, one anemone was washed with 500 µL of f/2 media + K/A/S (Kanamycin/Ampicillin/ Streptomycin: 50/100/50 µg/mL) and then crushed in fresh 500 µL of f/2 media + K/A/S using a glass tissue grinder (Duran Wheaton Kimble, USA) to keep the algal cells intact. This was filtered through a 40 µm nylon mesh sterile cell strainer (Fisherbrand, Fisher Scientific, USA), diluted in 15 mL of f/2 media + K/A/S and subsequently transferred to liquid culture flasks (225 cm^2^ Nunc Cell Culture Treated EasYFlask, Thermo Scientific) with 5 mL of the diluted fresh extract and 150 mL of f/2 media + K/A/S + GeO_2_ (4.47 µg/mL) saturated solution each to prevent diatom and bacteria growth. Presence of algal cells was checked under a fluorescence microscope (Leica DM3000 B inverted phase contrast microscope, Leica Microsystems GmbH, Germany). Moreover, solid agar f/2 + K/A/S plates were inoculated with the same fresh extract (normal concentration, 5-fold and 25-fold dilution) so single colonies could be grown and isolated for further experiments. After 10 days, 15 to 20 single colonies were picked from each plate, grown in 1.5 mL Eppendorf tubes with 300 µL of f/2 media + K/A/S and transferred to culture flasks (75 cm^2^ Nunc Cell Culture Treated EasYFlask, Thermo Scientific), as described before. The K/A/S treatment was performed every 10 subcultures to clear the bacterial load and, verified by growth on marine broth and inspected under the microscope. The above was performed under a flow hood (NuAire, Plymouth, MN, USA) to avoid environmental contamination. All liquid and solid cultures were kept in an incubator (Model I-22LLVL, Percival Scientific, USA) at 29 °C on a 12:12 h light:dark cycle (100 µmol photons/ m^2^ s of photosynthetically active radiation).

**Methods S2: DNA extraction, protein content and symbiont counts**

Genomic DNA for Symbiodiniaceae typing was isolated from 10 mL of liquid culture that was centrifuged at 1500 rcf for 10 min, where the supernatant was removed and the pellet was resuspended in 0.5 mL of cell lysis buffer from the DNeasy Plant Mini Kit (Qiagen, Hilden, Germany) in a 2 mL screw top Eppendorf tube. The cells were then homogenized using glass beads in a Tissue Lyser II (Qiagen, Hilden, Germany) set at 30 Hz for 30 s. The homogenate was spun down at 16 000 rcf for 10 s and the supernatant was taken for all downstream processing steps as according to the manufacturer’s instruction manual for the DNeasy Plant Mini Kit. This was done for each strain, from which three technical replicates were independently DNA-extracted.

For Aiptasia samples, a tentacle from each anemone was removed to extract DNA with the Chelex 100^®^ (Bio-Rad) resin method [2]. The tissue was vortexed with Chelex slurry for 20 s and briefly spun down in a picofuge. Samples were then incubated for 20 m in at 99 ºC, vortexed again for 20 s and spun down at 16 000 rcf for 2 min. Supernatant was later used as template for PCR. Each anemone was then crushed in 500 µL of cell lysis buffer (200 mM TRIS pH 7.5, 2 M NaCl, 0.1 % Triton 20 %) and two aliquots of 100 µL and 400 µL were immediately snap-frozen in liquid nitrogen and stored at -20 °C for further analyses of protein content and symbiont concentrations, respectively. Total host protein content was quantified with a Micro BCA Protein Assay Kit (Thermo Scientific, USA) using triplicates of 150 µL of 15x-diluted tissue slurry as per manufacturer instructions. Protein concentrations were measured at 562 nm absorbance using a SpectraMax Paradigm Multi-Mode Detection Platform (Molecular Devices, CA, USA). Symbiont cell counts were done using a flow cytometer (BD LSRFortessa, BD Biosciences, USA). Tissue homogenates were spun down at 14 000 *g* for 5 min, the supernatants were removed, and the pellet was re-suspended in phosphate buffered saline solution. The latter was performed twice before filtering the sample through a 40 µm nylon mesh sterile cell strainer (Fisherbrand, Fisher Scientific, USA). Cells were excited at a wavelength of 488 nm and fluorescence emission was recorded at 695/40 nm. Symbiont cell densities were quantified in triplicate measurements (20 μL each) based on forward-scattered light and chlorophyll autofluorescence signals of recorded events.

**Methods S3: ITS2 library preparation**

Each PCR reaction was run with Qiagen Multiplex PCR Kit (Qiagen, Germany) and 10 μM primers in a final reaction volume of 15 μL. Thermal cycling conditions of 15 min at 95 °C, followed by 30 cycles of 30 s at 95 °C, 90 s at 56 °C and 30 s at 72 °C, with a final extension step of 10 min at 72 °C were used for amplification. For each sample, PCR products were then run on a 1 % agarose gel electrophoresis pooled and cleaned using ExoProStar 1-step (GE Healthcare, Little Chalfont, UK). Subsequently, indexing was performed using the Nextera XT Index Kit (Illumina, CA, USA) according to the manufacturer’s instructions and followed by sample normalization and final library pooling. A SequalPrep Normalization Plate Kit (Invitrogen, Thermo Fisher Scientific, USA) was used to do the normalization, avoiding the more labor-intensive process of quantifying and aliquoting each individual sample. The final pooled library was quantified on a BioAnalyzer (Agilent Technologies, CA, USA) and sequenced at 7 pM with 20 % phiX on the Illumina MiSeq, 2 × 300 bp end version 3 chemistry according to the manufacturer's specifications at the Bioscience Core Lab in KAUST, Saudi Arabia.

**Methods S4: SymPortal analyses**

Although the ITS2 marker has been widely used to genetically characterize Symbiodiniaceae taxa, the multicopy nature of this gene complicates its use. A single cell can contain hundreds to thousands of rRNA gene copies [3]. Thus, variations among copies can give rise to an extraordinarily high intragenomic ITS2 diversity. SymPortal [4] makes use of this intragenomic diversity (which it refers to defining intragenomic variants or DIVs) to identify genotype representatives (type profiles) of putative Symbiodiniaceae taxa. However, despite being capable of resolving genetic delineations using the ITS2 marker at a finer level not possible before (at least without the use of additional markers), SymPortal’s approach has some inherent limitations (see [4]). Particularly, that if Symbiodiniaceae taxa co-occur in a sufficient number of samples (SymPortal currently uses an arbitrary support threshold of 4, Ben Hume *personal communication*), the ITS2 sequences returned from several taxa may be reports as being representative of a single taxon (i.e. super type). Though this is not typical for corals, as they usually harbor only one dominant symbiont species from a given genus, it seems to be the case for our Aiptasia samples (i.e. artificially created organisms). Indeed, preliminary analyses revealed the artifactual genotype A1/A4 (i.e. presence of both A1 and A4 taxa). This prompted us to perform an *in silico* analysis to test the power of resolution of SymPortal to correctly identify taxa across samples (see below).

Sequences of each (pure) symbiont culture were randomly subsampled in order to test different mixes in ratios of 75:25, 50:50 and 25:75: (i) SSA01:SSB01, (ii) SSA01: RS-A, (iii) SSA01:RS-B, (iv) SSB01:RS-A, (v) SSB01:RS-B, (vi) RS-A:RS-B and (vii) SSA01:SSB01:RS-A:RS-B (25:25:25:25). When analyzing these at the levels of type profile (Fig. S1a) and majority ITS2 sequence (Fig. S1b) it became apparent that SymPortal could not resolve mixes containing different proportions of SSA01 and RS-A, and SSB01 and RS-B (see *). Notably, the *Brevioulum* taxa exhibited distinct ITS2 type profiles (Fig. S1a) for the Hawaii and Red Sea strain and so we defined them as B1 and RS-B1, respectively (Fig. S1b).

We thus analyzed data prior to the minimum entropy decomposition (MED) analyses; that is, before SymPortal consolidates a high sequence diversity into a smaller set (based on biologically informative sequence positions). To do this, we first identified the diagnostic sequences of each symbiont strain. A1and A4 were distinct and symptomatic sequences of RS-A and SSA01, respectively, and B1 was common to SSB01 and RS-B. The variant 1369_B, on the other hand, was only present (with the highest abundance) in SSB01 and completely absent in RS-B. Thus, by using its abundance in a given sample, we could calculate the relative proportions of B1 and RS-B. For example, the minor copy 1369_B was present in SSB01 at ~ 0.00655 relative abundance (see Table S1). Any sample with the sequence B1 was then checked for 1369_B such that any deviation from the 1369_B:B1 ratio (~ 0.01268 for SSB01) would indicate how much RS-B was present. Relative abundances of all taxa were normalized to the abundance of their respective diagnostic ITS2 sequence and adjusted for slight deviations in the ratios so that the total sum was not higher than 1 (100%). This was done by correcting for ‘others’ so that if the sum was 105 %, for example, all values for that sample (i.e. row) were reduced by 5 %. Contrary, if the sum was below 100 % ‘others’ corresponded to sequences belonging to other than the four strains we tested here (i.e. *Cladocopium* and *Durisdinium* taxa which might have originated from cross-contamination while maintaining different Aiptasia in the same incubator and/or during PCR amplification; see methodological considerations in Discussion). Notably, the biggest error in absolute abundance was ~ 3.5 % (e.g. SSA01:SSB01 (25:75) mix was 26.86:71.45, see Table S1). Regardless, results of this analysis showed mixes were well resolved (Figure S2).

**References**

1. Matthews JL, Sproles AE, Oakley CA, Grossman AR, Weis VM, Davy SK. Menthol-induced bleaching rapidly and effectively provides experimental aposymbiotic sea anemones (Aiptasia sp.) for symbiosis investigations. *J Exp Biol* 2016; **219**: 306.

2. Walsh PS, Metzger DA, Higuchi R. Chelex® 100 as a medium for simple extraction of DNA for PCR-based typing from forensic material. *BioTechniques* 1991; **10**: 506–513.

3. Thornhill DJ, LaJeunesse TC, Santos SR. Measuring rDNA diversity in eukaryotic microbial systems: how intragenomic variation, pseudogenes, and PCR artifacts confound biodiversity estimates. *Molecular Ecology* 2007; **16**: 5326–5340.

4. Hume BCC, Smith EG, Ziegler M, Warrington HJM, Burt JA, LaJeunesse TC, et al. SymPortal: A novel analytical framework and platform for coral algal symbiont next-generation sequencing ITS2 profiling. *Molecular Ecology Resources* 2019; **19**: 1063–1080.

**
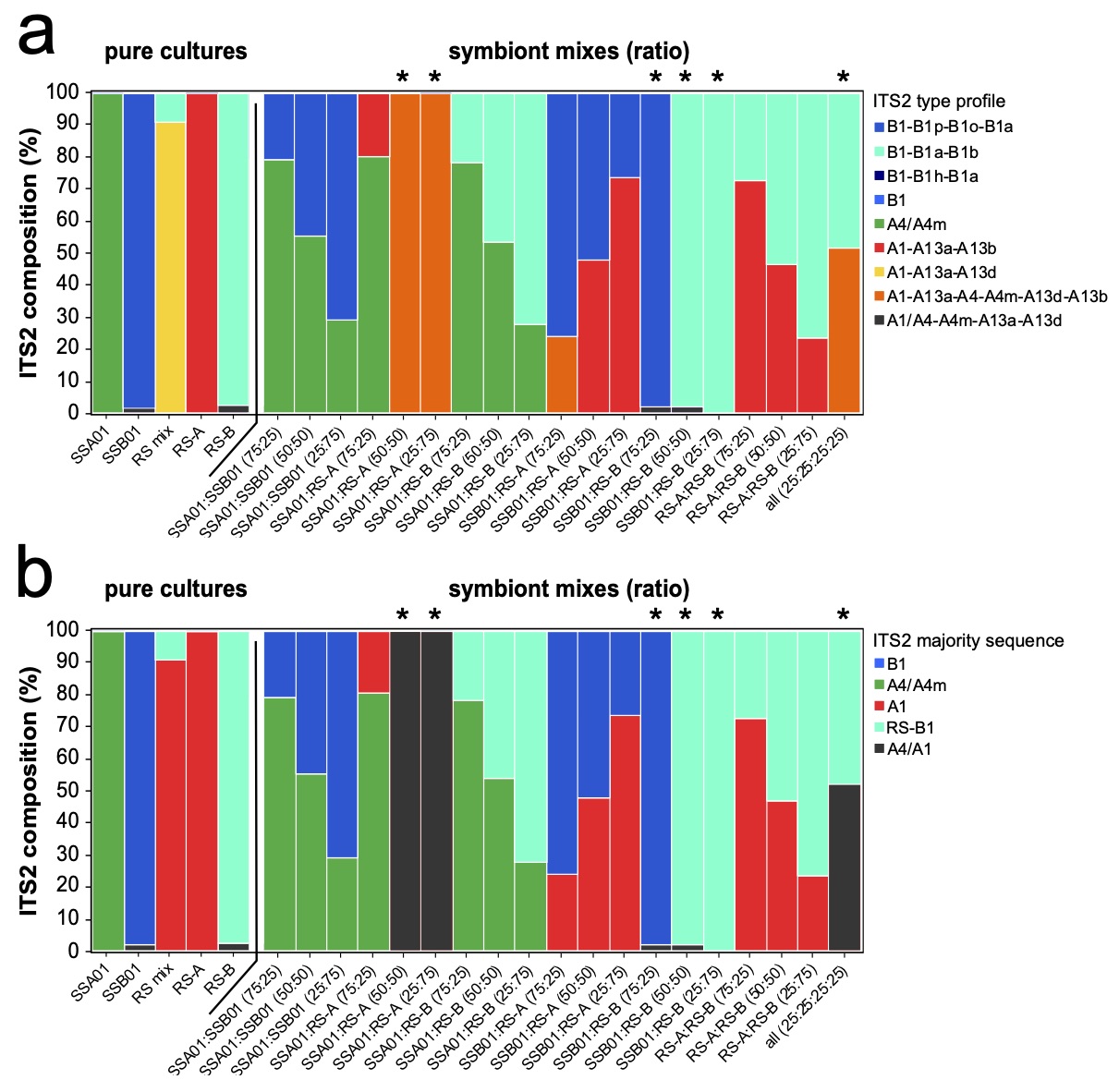
Figure S1.** Results from *in silico* analyses testing the power of SymPortal to resolve Symbiodiniaceae mixes at the level of **(a)** ITS2 type profiles and **(b)** majority sequence. Left panel shows the four symbiont strains (SSA01, SSB01, RS-A and RS-B) used to make the mixes. RS mix is the original Red Sea culture from which RS-A and RS-B taxa were isolated. Right panel shows all mixes and ratios tested. Notably, the mix all (25:25:25:25) has all four strains in equal proportions. Stars (*) indicate mixes that were not properly resolved (see Methods S4).

**Figure S2.** Results from *in silico* analyses testing the power of SymPortal to resolve Symbiodiniaceae mixes using the diagnostic sequence of each strain before the minimum entropy decomposition (MED) analyses. Left panel shows the four taxa (SSA01, SSB01, RS-A and RS-B) used to make the mixes. RS mix is the original Red Sea culture from which RS-A and RS-B strains were isolated. Right panel shows all mixes and ratios tested. The mix all (25:25:25:25) has all four strains in equal proportions. Stars (*) indicate mixes that could not be properly resolved before (Fig. S1) but that are now (see Methods S4).

**
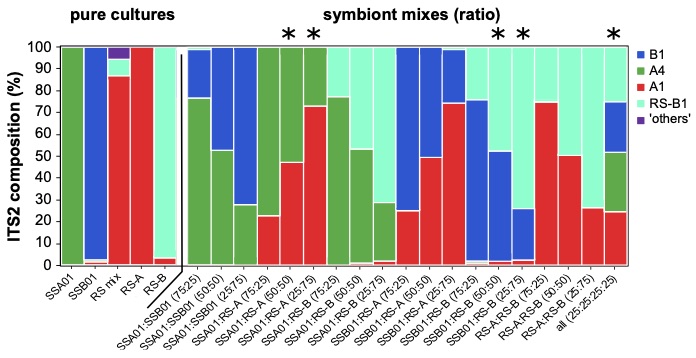
**

**
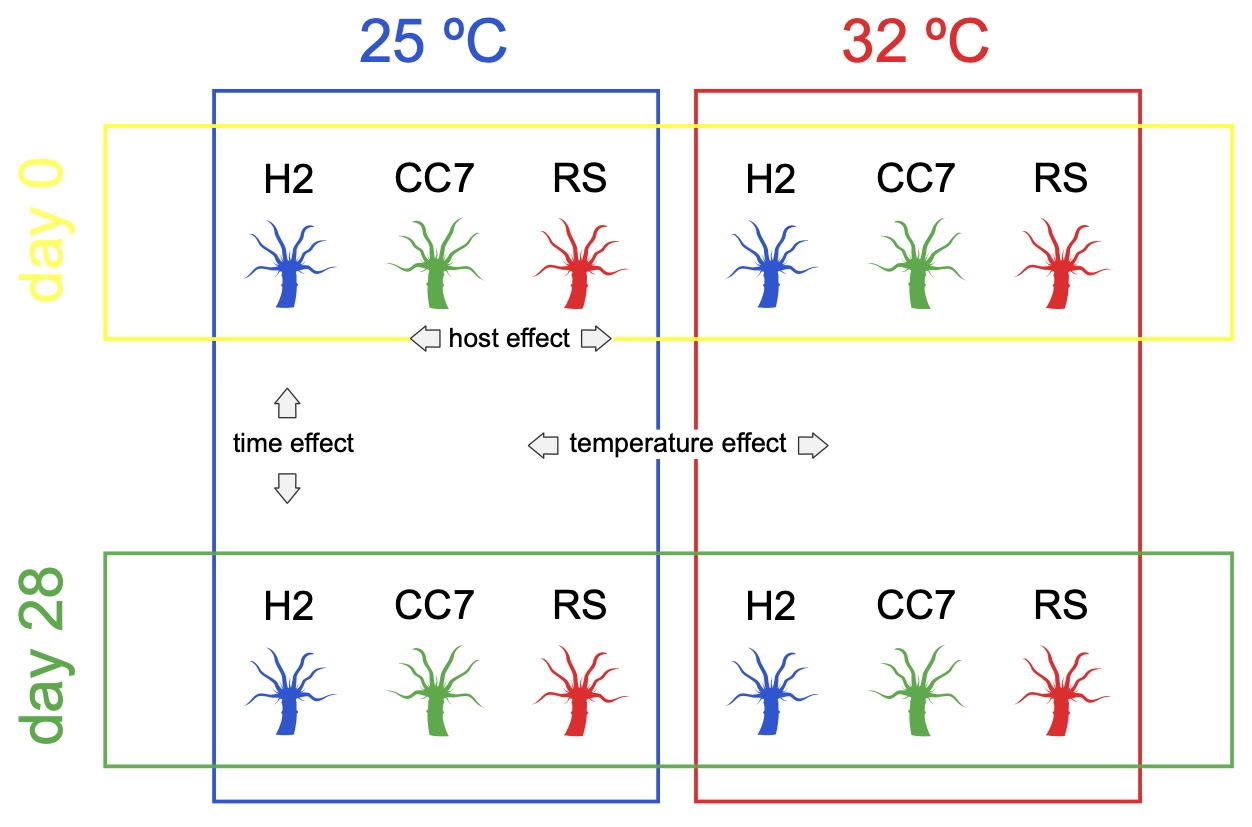
**

**Figure S3.** Illustration of experimental design shows Aiptasia from 3 different lineages (H2-Hawaii, CC7-North Carolina and RS-Red Sea) that were inoculated at 25 ºC and 32 ºC. Symbiont composition was assessed for each group (host x temperature) before (day 0) and after (day 28) being subjected to heat stress (32 ºC).

**
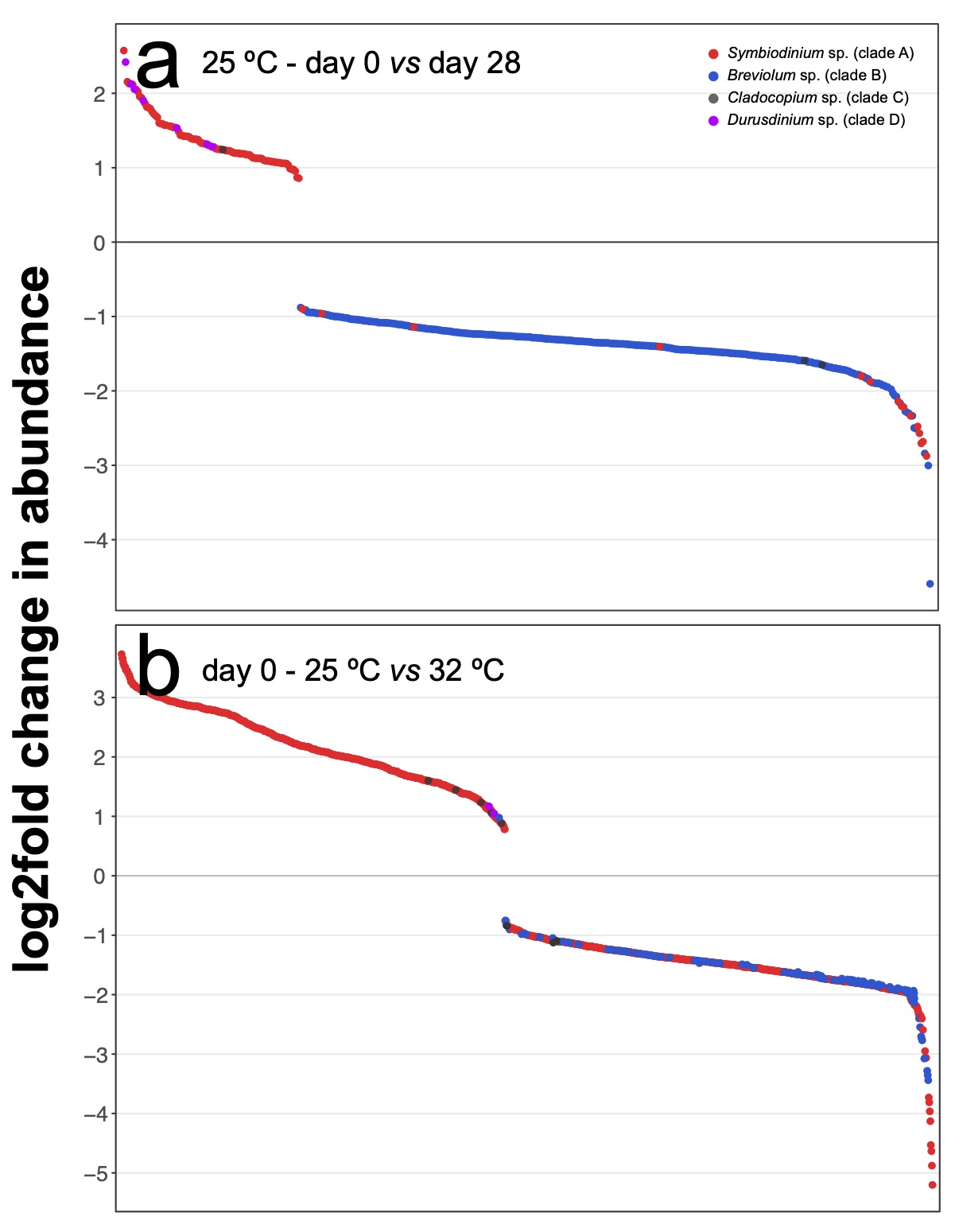
Figure S4.** Differentially abundant Symbiodiniaceae DIVs associated with Aiptasia from different treatments. Log2 fold change values indicate DIVs that show significant (*p* < 0.05) differential abundance at day 0 between holobionts from 32 ºC and 25 ºC (a) and after 28 days of heat stress compared to day 0 for individuals that originated at 25 ºC. Taxonomic classification (at the level of genus) is based on SymPortal’s annotations.
